# Indolent thyroid cancer: knowns and unknowns

**DOI:** 10.1186/s41199-016-0021-x

**Published:** 2017-01-11

**Authors:** Lewis D. Hahn, Christian A. Kunder, Michelle M. Chen, Lisa A. Orloff, Terry S. Desser

**Affiliations:** 1 Department of Radiology, Stanford University School of Medicine, 300 Pasteur Drive, H-1307, Mail code 5621, Stanford, CA 94305 USA; 2 Department of Pathology, Stanford University School of Medicine, Stanford, USA; 3 Department of Otolaryngology, Stanford University School of Medicine, Stanford, USA

**Keywords:** Thyroid, Cancer, Papillary, Differentiated, Indolent, Ultrasound, Molecular, Surveillance, microcarcinoma

## Abstract

Thyroid cancer incidence is rapidly increasing due to increased detection and diagnosis of indolent thyroid cancer, i.e. cancer that is likely to be clinically insignificant. Clinical, radiologic, and pathologic features predicting indolent behavior of thyroid cancer are still largely unknown and unstudied. Existing clinicopathologic staging systems are useful for providing prognosis in the context of treated thyroid cancer but are not designed for and are inadequate for predicting indolent behavior. Ultrasound studies have primarily focused on discrimination between malignant and benign nodules; some studies show promising data on using sonographic features for predicting indolence but are still in their early stages. Similarly, molecular studies are being developed to better characterize thyroid cancer and improve the yield of fine needle aspiration biopsy, but definite markers of indolent thyroid cancer have yet to be identified. Nonetheless, active surveillance has been introduced as an alternative to surgery in the case of indolent thyroid microcarcinoma, and protocols for safe surveillance are in development. As increased detection of thyroid cancer is all but inevitable, increased research on predicting indolent behavior is needed to avoid an epidemic of overtreatment.

## Background

Thyroid cancer incidence in the United States has more than tripled since 1973 [1–4]. Analysis of the National Cancer Institute’s (NCI) Surveillance, Epidemiology, and End Results (SEER) database over the period 1975–2009 by Davies and Welch suggests that virtually the entirety of the increase is due to greater detection and diagnosis of papillary thyroid carcinoma [2]. In principle, increased detection and treatment of early cancers is of benefit when associated with a decrease in cancer-related mortality; in reality, thyroid cancer-related mortality has not appreciably changed for at least 30 years because the vast majority of these cancers are indolent. The result instead has been increased surgery and procedure-related morbidity without a proven benefit in patient outcomes. By some estimates, the medical costs of thyroid cancer exceed 1.6 billion dollars a year in the U.S. [4, 5], and this figure is projected to more than double by 2030.

Although thyroid nodules are palpable in only 4–7% of adults [6, 7], the prevalence of thyroid nodules visible on ultrasound may be up to 67% [8]. Assuming an approximate 10% chance of any given thyroid nodule being malignant, this implies that millions of Americans have clinically occult thyroid cancer that is merely one ultrasound scan away from detection. Therefore, it has become crucial to not only identify thyroid cancer, but to also distinguish those thyroid cancers destined for indolent behavior from those likely to behave aggressively.

The goal of this review is to clarify what is known about identifying thyroid cancers that are likely to be clinically insignificant, and what questions still need to be investigated. We discuss the properties of indolent thyroid cancer as identified by clinical data, sonographic features, histopathology, and molecular markers in relation to the current guidelines and principles used to evaluate thyroid cancer. In addition, we discuss the management of indolent thyroid cancer.

### Clinicopathologic prognostic factors

Current guidelines for determining thyroid cancer prognosis are based on a variety of staging systems that classify tumors based on similar sets of clinicopathologic features. The primary features of TNM staging are size of tumor/extrathyroidal spread, extent of spread to lymph nodes, and the presence of distant metastases (Table 1). Large size of tumor, extrathyroidal extension, and the presence of distant metastases have separately shown consistent correlation with cancer-specific mortality [9–11]. Many (though not all) studies also support the association of nodal spread at presentation with mortality [10, 11]. Unlike other cancers, thyroid TNM staging also incorporates patient age, as advanced age confers poor prognosis for both mortality and recurrence [12]. Current TNM staging as proposed by the American Joint Committee on Cancer (AJCC) and Union for International Cancer Control (UICC) upstages some cancers in patients greater than 45 years of age. Recently, the hard cutoff of 45 has been challenged as mortality continues to increase with age, and the use of a nomogram to determine stage based on age as a continuous variable may yield improved prediction of prognosis [12]. Similar criticism could conceivably be directed at other variables of the TNM system. Nonetheless, TNM staging remains a well-validated tool for initially stratifying patients into four categories differing by cancer-specific mortality [9, 13]. Another commonly used system is the MACIS system developed at the Mayo clinic (Table 2) which calculates a score based on a similar set of features that also reliably stratifies patients into four prognostic categories [13]. One recent Danish prospective study followed a cohort of 1350 patients with papillary thyroid carcinoma over a median time of 7.9 years to assess the accuracy of the 5th edition of TNM, MACIS, and additional staging systems. [13] TNM staging and MACIS both achieved high statistical significance in distinguishing stages by cancer specific mortality, thus validating their use in a modern cohort.

**Table 1:** TNM staging for differentiated cancers (papillary and follicular)

Primary Tumor (T)	
TX	Primary tumor cannot be assessed
T0	No evidence of primary tumor
T1	Tumor 2 cm or less in greatest dimension limited to the thyroid
T1a	Tumor 1 cm or less, limited to the thyroid
T1b	Tumor more than 1 cm but not more than 2 cm in greatest dimension, limited to the thyroid
T2	Tumor more than 2 cm but not more than 4 cm in greatest dimension limited to the thyroid
T3	Tumor more than 4 cm in greatest dimension limited to the thyroid or any tumor with minimal extrathyroid extension (e.g., extension to sternothyroid muscle or perithyroid soft tissues)
T4a	Moderately advanced disease: Tumor of any size extending beyond the thyroid capsule to invade subcutaneous soft tissues, larynx, trachea, esophagus, or recurrent laryngeal nerve
T4b	Very advanced disease Tumor invades prevertebral fascia or encases carotid artery or mediastinal vessels
Regional Lymph Nodes (N)	Regional lymph nodes are the central compartment, lateral cervical, and upper mediastinal lymph nodes.
NX	Regional lymph nodes cannot be assessed
N0	No regional lymph node metastasis
N1	Regional lymph node metastasis
N1a	Metastasis to Level VI (pretracheal, paratracheal, and prelaryngeal/Delphian lymph nodes)
N1b	Metastasis to unilateral, bilateral, or contralateral cervical (Levels I, II, III, IV, or V) or retropharyngeal or superior mediastinal lymph nodes (Level VII)
Distant Metastasis (M)	
M0	No distant metastasis
M1	Distant metastasis
Age less than 45 years	
Stage I	Any T Any N M0
Stage II	Any T Any N M1
Age greater than 45 years	
Stage I	T1 N0 M0
Stage II	T2 N0 M0
Stage III	T3 N0 M0 T1 N1a M0 T2 N1a M0 T3 N1a M0
Stage IVA	T4a N0 M0 T4a N1a M0 T1 N1b M0 T2 N1b M0 T3 N1b M0 T4a N1b M0
Stage IVB	T4b Any N M0
Stage IVC	Any T Any N M1

Used with permission of the American Joint Committee on Cancer (AJCC), Chicago, Illinois. The original and primary source for this information is the AJCC Cancer Staging Manual, Seventh Edition (2010) published by Springer Science + Business Media

**Table 2:** MACIS scoring and staging

MACIS Score components	
Metastases	3 if distant spread
Age	3.1 (if age <40 years) or 0.08 × age (if age ≥ 40 year)
Completeness of Resection	1 if incompletely resected, 0 otherwise
Invasion (local)	1 if locally invasive, 0 otherwise
Size	0.3 × tumor size (cm maximum diameter)
MACIS stage	MACIS score threshold
Stage 1	<6
Stage 2	6–6.99
Stage 3	7–7.99
Stage 4	≥8

TNM staging is limited in its prediction of disease recurrence, and therefore the American Thyroid Association (ATA) provides a separate Initial Risk Stratification System to divide patients with well differentiated thyroid cancer into three risk groups differing by likelihood of recurrence. In addition to incorporating TNM features, these guidelines also incorporate some factors related to initial treatment. High-risk features include incomplete tumor resection or postoperative serum thyroglobulin levels suggestive of distant metastases. Intermediate-risk features include RAI-avid metastatic foci of the neck on post-treatment whole-body RAI scan, clinical N1, microscopic perithyroidal invasion and others. Low risk features include papillary cancers with no local or vascular invasion, no local or distant metastases, and complete macroscopic tumor resection. Initial Risk Stratification System also takes into account histopathology, and defines patients with aggressive histologies such as tall cell, insular, or columnar cell variants, or vascular invasion, as being at higher risk of recurrence [14, 15].

Despite their widespread use, TNM staging for mortality and ATA risk stratification for recurrence demonstrate relatively low proportion of variance explained (PVE), a statistical measure that can be used to assess prognosis classification systems. This likely relates to an extremely limited incorporation of treatment response. Recent studies have proposed and validated dynamic systems of risk stratification, in which patients are reassessed following each round of treatment using a combination of imaging findings and thyroglobulin, which dramatically increased PVE for recurrence from 25–34% to 62–84 [16, 17]. It is important to recognize, however, that risk stratification would ideally be performed before any major intervention, and that prediction of indolent behavior in the absence of treatment is a distinct goal from determining prognosis after treatment. Any thyroid cancer with evidence of regional or distant spread could not be considered indolent and would certainly require intervention (though this may require further evaluation in the future). However, SEER data from 1973 to 2013 indicate that 68% of thyroid cancers are confined to the thyroid at diagnosis [18], and other studies show that an even higher percentage may be associated with micrometastases that are not clinically significant (14). The task, then, is to identify which of these majority of well-differentiated thyroid cancers are indolent.

Small tumor size in itself is a good first clue, as the vast majority of thyroid microcarcinoma, defined as thyroid cancer less than one centimeter in size, is indolent. This is well demonstrated by autopsy studies which found a prevalence of thyroid microcarcinoma 10–100 times greater than the prevalence of microcarcinoma which presents clinically [19]. In keeping with the hypothesis that increased thyroid cancer detection is responsible for increased thyroid incidence, microcarcinomas now account for a bulk of newly detected thyroid cancer. Microcarcinomas represented 39% of all cancers identified in the United States from 2008 to 2009, versus 25% in 1988–89 [2]. The relative proportion of cancers larger than 2 cm decreased from 42 to 33% over the same period. In an effort to stave off a diagnostically induced epidemic of thyroid microcarcinoma, current guidelines by the ATA [14] and Society of Radiologists in Ultrasound (SRU) [20] recommend abstaining from fine needle aspiration of thyroid nodules less than a centimeter in size; a small thyroid nodule may be considered benign or indolent until proven otherwise. Appropriate follow-up is then determined on the basis of the presence or absence of worrisome sonographic features within the thyroid nodule and the surrounding neck.

### Sonographic prognostic factors

**Fig. 1:**
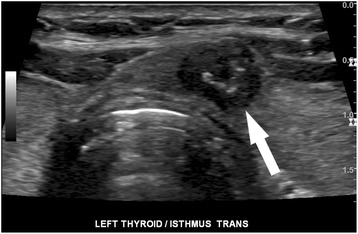
High suspicion pattern. Transverse grayscale sonographic image of the thyroid at level of isthmus shows a hypoechoic, irregularly marginated thyroid nodule containing microcalcifications (*arrow*). This is a “high suspicion” sonographic pattern in the 2015 ATA guidelines, with an estimated risk of malignancy of >70–90%. Fine-needle aspiration of this nodule showed papillary thyroid carcinoma

**Fig. 2:**
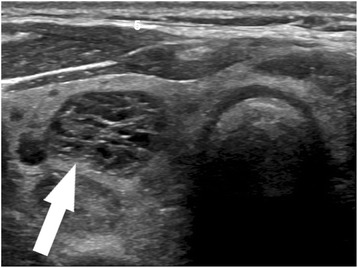
Very low suspicion pattern. Transverse grayscale sonographic image of the thyroid at level of thyroid isthmus shows a nodule in the right lobe (*arrow*) with a “spongiform” pattern. Note the innumerable tiny cystic spaces characteristic of this pattern

**Fig. 3:**
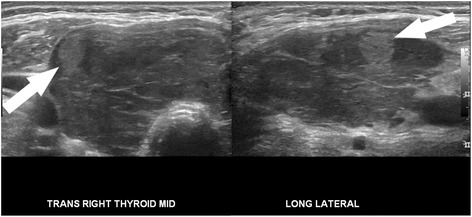
Very low suspicion pattern. Transverse and longitudinal sonographic images of the right lobe of the thyroid show an echogenic nodule (*arrow*) against a background of hypoechoic, enlarged thyroid with curvilinear echogenic bands. These features of the background thyroid are characteristic of Hashimoto’s thyroiditis. The echogenic nodule is a benign “white knight” nodule, representing a regenerative nodule

Ultrasound would be an ideal tool for prediction of indolent cancer, given that diagnosis of thyroid cancer almost always begins with sonographic evaluation of a thyroid nodule. An array of sonographic features has been used to describe thyroid nodules and identify those with features suspicious for malignancy. These features include microcalcifications, irregular borders, increased vascularity, taller-than-wide morphology, and marked hypoechogenicity (Fig. 1) [21, 22], though no single feature individually has proved to be very sensitive or specific. Conversely, a few sonographic patterns have been reliably associated with benignity in thyroid nodules, including a “spongiform” appearance (Fig. 2), an echogenic appearance in the setting of Hashimoto’s thyroiditis termed a “white knight” nodule (Fig. 3), a “giraffe skin” appearance, and a purely cystic nodule containing colloid without flow [23–25]. Elastography, a relatively new sonographic technique which assesses nodule stiffness, appears to correlate with malignancy, but further validation and wide-spread adoption will be required before stiffness measurements can be incorporated into routine thyroid nodule evaluation [26].

Recent efforts have been made to standardize reporting of thyroid nodule findings and help stratify risk of malignancy [22], some of which have been described as a Thyroid Imaging Reporting and Data System [27, 28] (TI-RADS) analogous to BI-RADS for cataloguing breast masses. The American College of Radiology is also in the process of developing its own TI-RADS system and has proposed a lexicon for describing thyroid nodules [29]. The ATA makes specific recommendations for biopsy based on different risk categories of sonographic features paired with lesion size (Table 3). While these efforts are important steps toward standardization and determining management according to current guidelines, to date most work has involved the binary classification of nodules as benign or suspicious for malignancy. Future research must add nuance to this characterization question and aim to identify which sonographic features of nodules predict not just malignancy, but an *aggressive* thyroid cancer versus an *indolent* thyroid cancer versus a benign thyroid nodule.

**Table 3:** Sonographic Patterns, Estimated Risk of Malignancy, and Fine-Needle Aspiration Guidance for Thyroid Nodules, reproduced from 2015 American Thyroid Association guidelines [14]

Sonographic pattern	US features	Estimated risk of malignancy, %	FNA size cutoff (largest dimension)
High suspicion	Solid hypoechoic nodule or solid hypoechoic component of a partially cystic nodule with one or more of the following features: irregular margins (infiltrative, microlobulated), microcalcifications, taller than wide shape, rim calcifications with small extrusive soft tissue component, evidence of ETE	>70–90	Recommend FNA at ≥1 cm
Intermediate suspicion	Hypoechoic solid nodule with smooth margins without microcalcifications, ETE, or taller than wide shape	10–20	Recommend FNA at ≥1 cm
Low suspicion	Isoechoic or hyperechoic solid nodule, or partially cystic nodule with eccentric solid areas, without microcalcification, irregular margin or ETE, or taller than wide shape.	5–10	Recommend FNA at ≥1.5 cm
Very low suspicion	Spongiform or partially cystic nodules without any of the sonographic features described in low, intermediate, or high suspicion patterns.	<3	Consider FNA at ≥2 cm Observation without FNA is also a reasonable option
Benign	Purely cystic nodules (no solid component)	<1	No biopsy

A small number of studies have begun to investigate sonographic prediction of aggressive tumor behavior. Most assess the association of findings on pre-operative ultrasound with extracapsular extension and lymph node spread at the time of surgery (Table 4). Capsular invasion/abutment correlates with extrathyroidal extension [30–33], a characteristic of more locally advanced disease and poorer prognosis. Methods for pre-operative sonographic evaluation of capsular invasion differ. One study found that capsular abutment, bulging of the thyroid contour, and loss of the echogenic thyroid capsule have excellent predictive value for excluding or detecting extracapsular extension [33]. Tumor vascularity and microcalcifications have shown variable correlation with extrathyroidal extension and nodal spread [30, 32, 34].

**Table 4:** Representative literature on sonographic prediction of thyroid cancer prognosis

Author	Year	Number of cases and histologic subtype	Size range	Prognostic measure	Sonographic parameters studied	Selected results
Cappelli et al. [53]	2007	484 PTC	<1.0 cm to > 4.0 cm	Recurrence of disease or death due to thyroidcancer	Blurred margins, presence of calcifications, intranodular vascularity, hypoechogenicity, multifocality, extracapsular growth	Among investigated sonographic parameters, only intranodular flow associated with unfavorable outcome
Du et al. [34]	2015	177 PTC 3 follicular 6 medullary	N/A	LN mets	Size, peak systolic velocity, pulsatility index, resistive index, multifocality, bilateral vs. unilateral, nodule border, edge irregularity, halo, solid/cystic vs. solid, uniformity of echogenicity, echogenicity, microcalcifications, flow grade, capsular invasion	Large size, percent contact with thyroid capsule, microcalcifications, flow grade 3–4 (graded from 0–4), resistive index >0.654, peak systolic velocity > 24.5 cm/s associated with LN mets. Additional categories not associated with LN mets.
Fukuoka et al. [35]	2015	480 PTC in 384 patients	<1.0 cm	Increase in tumor size ≥3 mm (prospective trial)	Calcification pattern, tumor vascularity	Macroscopic/rim calcifications and poor vascularity on most recent follow-up associated with non-progression of disease. These features were also strongly associated with advanced age.
Gweon et al. [54]	2016	397 PTC	3–35 mm	ETE LN mets	Tumor composition, echogenicity, margins, calcifications, shape, TI-RADS category (Kwon classification), size	Size associated with ETE. Size, microcalcifications associated with LN mets. All additional categories not associated with ETE or LN mets
Kamaya et al. [33]	2015	62 PTC	>1.0 cm	ETE	Capsular abutment, contour bulging, vascularity beyond capsule, loss of echogenic capsule	Capsular abutment 100% sensitive for extracapsular extension Loss of echogenic capsule was best predictor of ETE.
Kim et al. [30]	2011	354 PTC	≤2 cm	ETE LN mets	Size, shape, margin, echogenicity, calcification, vascularity, contact with capsule	Size >0.5 cm, marked hypoechogenicity, contact with capsule associated with ETE. Marked hypoechogenicity associated with LN mets. Additional factors were not predictive.
Lai et al. [55]	2016	367 PTC	≤1.0 cm	ETE LN mets	Size, shape, length/width ratio, border, peripheral halo, echogenicity, cystic change, calcification (any), vascularity, presence of Hashimoto’s thyroiditis	Size associated with LN mets and ETE. Calcification (any), multifocality associated with LN mets only for microcarcinoma > 5 mm. Additional features were not associated with LN mets; no US features associated with LN mets for microcarcinoma < 5 mm.
Lee et al. [31]	2014	568 PTC	3–49 mm	ETE	Size, lesion location, echogenicity, (LN stage), % abutment of thyroid capsule, capsular protrusion	Size, thyroid capsular protrusion, % abutment of thyroid capsule are all associated with ETE.
Zhan et al. [32]	2012	155 PTC	<10 mm to greater than 40 mm	LN mets	Size, shape, border, margin, halo, internal architecture, echogenicity, homogeneity of echotexture, calcification, contact between nodule border and thyroid, vascularity, peak systolic velocity, pulsatility index, resistive index	Size, contact percentage, combined microcalcifications/ macrocalcifications, increased vascularity, high resistive index difference associated with LN mets. No association seen with other parameters.

*PTC* papillary thyroid carcinoma, *ETE* extrathyroidal extension, *LN* mets, LYMPH node metastases

Determination of sonographic features associated with extracapsular or nodal spread at surgery may be suggestive of aggressive behavior but is not equivalent to determining features associated with progression in potentially indolent thyroid cancers isolated to the thyroid gland at diagnosis. One study directly examining this question was published in 2015 by Fukuoka and colleagues [35]. This group followed 480 papillary microcarcinomas over a mean of 6.8 years and sought to determine features that predicted disease progression as defined by an increase in size ≥3 mm. Interestingly, sonographic patterns of microcarcinomas changed over time; nodules that developed coarse or rim calcifications tended to have less risk of progression of disease, and those with poor vascularity also had less chance of progression. Overall risk of progression was best correlated with calcification pattern and vascularity on the most recent follow-up.

Investigation into the sonographic features predicting tumor aggressiveness are still in their early stages. Many existing studies retrospectively examine thyroid cancer imaged immediately before surgery, and often those studies were performed merely to localize a nodule for fine-needle aspiration, rather than with a focused high-resolution technique designed for precise local staging. Additional prospective studies are needed to examine sonographic features that correlate with poor prognosis; as further discussed below, this can safely be performed for microcarcinomas. For larger cancers in which surveillance would currently be inappropriate, new studies are needed that correlate postsurgical natural history with preoperative sonographic analysis, which may provide additional insight into which sonographic features suggest aggressive malignancy. Moreover, existing studies significantly differ in their use of descriptive features. The development of a standardized lexicon such as TI-RADS should facilitate larger, multi-institutional research to tease out which, if any, sonographic features might be most predictive of clinical behavior. Such studies would ultimately inform the future modification of TI-RADS and guidelines for clinical management by recommending immediate surgery, surveillance, or no follow-up.

### Molecular prognostic factors

The current classification of thyroid neoplasms (and non-neoplastic thyroid lesions) is based on histopathology [36]. While the existing classification is exceedingly useful, it is apparent that additional refinements are necessary to ultimately arrive at a classification that clearly separates biologically (or at least clinically) distinct entities. As it is clear that the great majority of “carcinomas” are clinically indolent, even the most basic distinction between “benign” and “malignant” requires reappraisal.

As in other areas in oncology, thyroid pathology has generally been focused on the identification of features that predict aggressiveness, within the accepted “malignant” categories of papillary and follicular thyroid carcinoma, the most common histologic types. While uncommon morphologic subtypes of papillary thyroid carcinoma (PTC) have been associated with poor prognosis, these often correlate with staging parameters. The alternative approach (identifying features that predict indolence) is less common but clearly necessary, as in the recent call to rename encapsulated follicular-variant papillary thyroid carcinoma to “encapsulated follicular neoplasm with papillary-like nuclear features” [37], reflecting the fact that, like follicular adenomas, this tumor lacks malignant potential.

Classification and nomenclature are likely to undergo many more changes in the future as our names for tumors are informed to a greater and greater extent by genetic data. The relationship between mutations or gene expression profiles and histologic patterns is very complex, but a clearer picture is emerging thanks to efforts like the Cancer Genome Atlas (TCGA) study on PTC [38]. It appears that like lung adenocarcinoma, PTC is typified by a number of “driver” mutations that are mutually exclusive. Conventional-type papillary thyroid carcinomas are largely driven by the *BRAF* V600E mutation, while follicular variant PTCs show a high frequency of *RAS* mutations. Follicular neoplasms (both follicular adenomas and follicular carcinomas), also show a high frequency of *RAS* mutations [39], raising the question of whether follicular variant PTC is a type of PTC (by virtue of its nuclear features) or a type of follicular carcinoma (by virtue of its molecular underpinnings). Protein kinase fusion genes drive a significant minority of PTC cases, the spectrum of which overlaps heavily with fusions seen in lung adenocarcinoma. Both *BRAF* mutations and kinase fusions represent opportunities for targeted therapy, an active area of inquiry in advanced disease.

Thus far, clinical molecular testing in thyroid neoplasia has largely focused on improving the diagnostic yield of the fine needle aspiration (FNA) biopsy, the current mainstay for the diagnosis of thyroid nodules. FNA is a very useful technique, but a significant minority of cases are returned with non-definitive diagnoses, according to the Bethesda System for Reporting Thyroid Cytopathology [40], leading to uncertainty and anxiety. Furthermore, the proportion of cases receiving these diagnoses varies considerably by institution (and correspondingly, so does the incidence of cancer within this group) [41].

Most of the molecular assays currently in regular use were intended to reduce this uncertainty, leading to more appropriate surgical management. Unfortunately, no one of these tests currently has both high sensitivity and specificity. Afirma® (Veracyte Inc., South San Francisco CA) is a commercial gene expression classifier intended to “rule out” cancer after an atypical FNA result. For thyroid nodule FNAs interpreted as atypical or suspicious, the test had a published negative predictive value (NPV) of 85-95%, with the NPV decreasing in categories with a higher prevalence of cancer (for example “suspicious” versus “atypia of undetermined significance”) [42].

Mutational profiling is another method being used. This approach is exemplified by ThyroSeq®, which was developed at the University of Pittsburgh. This assay tests for a number of mutations and fusion genes either known to occur in cancers or only in benign thyroid neoplasms. The assay has been expanded several times to keep pace with newly discovered mutation types, but it is expensive and still has sensitivity and specificity only in the 90% range [43].

The molecular correlates of indolence versus aggressiveness are not well known, and almost all effort in this area has been expended towards identifying markers of aggressiveness. Some evidence suggests that the *BRAF* V600E mutation is associated with relatively poorer prognosis, although overall mortality is very low even in the *BRAF* V600E-positive group [44]. However, the TCGA data demonstrate that there is great biologic heterogeneity even within *BRAF* V600E-positive PTC [38]. *TERT* promoter mutations may also confer a more aggressive phenotype, and patients with both *BRAF* V600E and *TERT* promoter mutations appear to have a higher rate of aggressive disease, even after controlling for other clinicopathologic factors [45]. Although other alterations (such as *TP53* mutations) are associated with high-risk cancers, such tumors tend to be histologically poorly-differentiated or high stage. Aside from the weak association of *BRAF* V600E with risk, broad molecular subtypes (in terms of driver mutations or gene expression profiling) all contain a mixture of risk groups, and clear molecular categories without the potential for clinically aggressive disease have not yet emerged.

We are still establishing the playing field when it comes to the molecular basis of thyroid cancer. As sequencing technology becomes more and more available, the true molecular landscape of these neoplasms will emerge in all its complexity. Additional studies carefully correlating histopathologic and molecular features are necessary, including studies on benign thyroid neoplasms, which are currently poorly understood and doubtless hold important lessons on the nature of their malignant cousins. Hopefully, such studies will enable the confident identification of those cases without a significant risk of progression, as well those cases that are especially aggressive.

### Surgical management versus active surveillance of indolent differentiated thyroid cancer

Identification of indolent thyroid cancer will ultimately inform the decision to pursue surgical management versus active surveillance. The 2015 ATA guidelines recommend either a total thyroidectomy or a thyroid lobectomy (depending on overall thyroid status, patient age, personal and family history, etc.) for thyroid cancers >1 cm and <4 cm without extrathyroidal extension and lymph node metastases. On the other hand, a total thyroidectomy is recommended for all patients with primary tumors >4 cm, extrathyroidal extension, nodal metastatic disease, or distant metastatic disease [14]. Any patients with lateral or central neck lymph node metastases are strongly recommended to have therapeutic lateral and/or central neck dissections. For isolated thyroid microcarcinoma (<1 cm), if surgery is performed, then a lobectomy is considered sufficient.

However, recent literature from Japan has suggested that active surveillance may be sufficient for thyroid microcarcinoma without aggressive features, i.e. those that are most likely to be indolent. Ito et al. observed 1235 patients with micropapillary carcinoma and reported that over the course of 10 years of observation, only 8% had tumors that increased by ≥3 mm and 3.8% had new nodal metastases. No patients died or developed distant metastases [46]. Another study by Oda et al. demonstrated that of 1179 patients under active surveillance over an 8 year period, 94 (8.0%) underwent surgery and for most of these patients (54%), the main reason for surgery was that patients changed their minds and preferred surgery over continued surveillance [47].

As discussed, there is no reliable set of clinical or pathologic features that can distinguish which patients with thyroid carcinomas will progress and develop clinically significant disease. In addition to determining which patients are the best candidates for active surveillance, further research is needed to clarify optimal methods for active surveillance, such as the frequency of surveillance ultrasound exams. Brito et al. suggest that inappropriate candidates for active surveillance include those with documented increase in tumor size of ≥3 mm, subcapsular location adjacent to the recurrent laryngeal nerve, evidence of aggressive features (extrathyroidal extension, new nodal metastases, aggressive cytology on FNA), younger patients, and those who are unlikely to be compliant with follow-up [48].

The cost-effectiveness of thyroid lobectomy versus active surveillance is highly patient dependent and the current data is inconclusive. Venkatesh et al [49] modeled that active surveillance was cost-effective for patients who associated surveillance with a health utility <0.01 below that of a thyroid lobectomy, while for the majority (79%) of simulations they modeled, a lobectomy was cost-effective relative to active surveillance due to the disutility associated with active surveillance. These authors acknowledge that their model was not designed to perform individual, patient-specific simulations, and that patients in whom the diagnosis of even indolent microcarcinoma is associated with a modest decrement in quality of life may benefit from surgery as the cost-effective strategy. Nor did this study address any comparison between total thyroidectomy and active surveillance, and limitation to thyroid lobectomy was an admitted oversimplification of the spectrum of variables. Lang and Wong [50] also conducted Markov modeling and determined that active surveillance was cost-effective regardless of patient age, complications, or rates of progression. However, their study did not attempt to address the quality of life impact of living with a cancer diagnosis, but focused specifically on the economic costs associated with treatment versus surveillance of thyroid cancer. Regardless, active surveillance is not without risks and these include the need for close follow-up, frequent exams, and the risk of disease progression and metastasis.

Thus, in order to address these risks, consideration of active surveillance needs to be a shared and informed decision-making process between patients and their clinicians. Active surveillance also requires consistent high quality ultrasound exams for the monitoring of not only thyroid nodules, but also potential lymph node metastases. In the United States, thyroid ultrasound exams are performed by sonographers, radiologists, surgeons, and endocrinologists and there is significant variability in the quality of ultrasonography, especially in consistently identifying smaller thyroid nodules and lymph node metastases. Brauer et al. reported that the interobserver variation in measurement of thyroid nodules on ultrasound is about 50% [51]. While the probability of experienced ultrasonographers identifying the same thyroid nodule is 90% for nodules >1.5 cm, only about a third of nodules <1 cm could be could be identified as the same structure by multiple ultrasonographers [51].

## Conclusions

Current data suggests that the epidemic of thyroid cancer in the United States is primarily an epidemic of diagnosis rather than disease: while incidence of thyroid cancers appears to have tripled between 1975 and 2009, there has been no corresponding change in mortality. A recently published meta-analysis of autopsy studies has further confirmed that the prevalence of thyroid cancer has not significantly changed [52]. Some of the determinants of aggressive behavior in thyroid cancer have been elucidated, but features associated with indolent behavior *per se* remain unclear. As active surveillance strategies begin to emerge as an alternative to surgery, future studies are needed to identify those sonographic features that augur an indolent clinical course. Genetic analysis may also prove useful as more markers emerge that predict not just aggressive behavior, but benignity and indolence as well. Even after identification of patients with tumor characteristics that correlate with excellent prognosis, additional work will be needed to study practical considerations such as cost or availability of high quality sonography which may exclude some patients from active surveillance. Only after addressing all of these aspects of the workup and management of thyroid cancer will a strategy emerge for treating thyroid cancer effectively while avoiding needlessly aggressive therapy.

## Abbreviations

AJCC: American Joint Committee on Cancer
ATA: American Thyroid Association
ETE: Extrathyroidal extension
FNA: Fine needle aspiration
LN mets: Lymph node metastases
NCI: National Cancer Institute
NPV: Negative predictive value
PTC: Papillary thyroid carcinoma
PVE: proportion of variance explained
SEER: Surveillance, Epidemiology, and End Results
SRU: Society of Radiologists in Ultrasound
TCGA: The Cancer Genome Atlas
TI-RADS: Thyroid Imaging Reporting and Data System
UICC: Union for International Cancer Control
